# Acupoint catgut embedding for improving HOMA-IR in patients with abdominal obesity and insulin resistance: a protocol for systematic review and network meta-analysis

**DOI:** 10.3389/fnut.2026.1828235

**Published:** 2026-05-08

**Authors:** Lang Hu, Tao Zhu, Ming Li, Zhihui Zhang, Yizhou Chen, Luyao Cai, Dan Yang, Jin Cui

**Affiliations:** 1 Guizhou University of Traditional Chinese Medicine, Guiyang, China; 2 Department of Acupuncture and Moxibustion, The First Affiliated Hospital of Guizhou University of Traditional Chinese Medicine, Guiyang, China

**Keywords:** abdominal obesity, acupoint catgut embedding, insulin resistance, network meta-analysis, protocol, systematic review

## Abstract

**Objective:**

Acupoint catgut embedding (ACE) shows promise for improving insulin resistance in abdominal obesity, but its comparative efficacy against other interventions remains unclear due to a lack of head-to-head trials. This study aims to systematically evaluate the efficacy and safety of ACE for reducing HOMA-IR in this population and to compare different interventions via network meta-analysis.

**Methods:**

We will conduct a systematic search of 13 electronic databases, covering the period from the inception of each database to March 2026. This study will include all randomized controlled trials evaluating acupoint catgut embedding for the treatment of abdominal obesity with concomitant insulin resistance. Two review authors will independently perform literature screening, data extraction, and assess the risk of bias of included studies using the revised Cochrane risk-of-bias tool (RoB 2). The primary outcome measure will be the homeostasis model assessment of insulin resistance (HOMA-IR) index. Secondary outcome measures will include fasting plasma glucose, fasting insulin, waist circumference, body mass index, lipid profile, inflammatory markers, and adverse events. Data synthesis will be performed using Stata 17.0 and R software. Conventional pairwise meta-analyses will be conducted using random-effects models. For network meta-analysis, we will adopt a frequentist framework using multivariate random-effects meta-analysis. The design-by-treatment interaction model will be used to assess global inconsistency, and node-splitting analysis will be performed to evaluate local inconsistency for any closed loops in the evidence network. The surface under the cumulative ranking curve (SUCRA) will be used to estimate the probability of each intervention being ranked as the most effective. The strength of evidence will be assessed using the Grading of Recommendations, Assessment, Development and Evaluations (GRADE) framework.

**Conclusion:**

This study will compare the efficacy of acupoint catgut embedding against lifestyle interventions, pharmacotherapy, and other acupuncture modalities in improving insulin resistance among patients with abdominal obesity. The findings aim to inform clinical decisions and optimize therapeutic strategies. Additionally, subgroup analyses based on embedding depth, treatment frequency, ethnicity, and insulin resistance severity will help identify the most effective regimens for specific patient subgroups, guiding personalized treatment.

**Systematic review registration:**

https://www.crd.york.ac.uk/prospero/, CRD420261331031.

## 1 Introduction

Abdominal obesity, characterized by excessive fat accumulation in the visceral region, is closely associated with insulin resistance (IR)—a pathological condition in which cells exhibit an insufficient response to normal insulin levels. The coexistence of abdominal obesity and insulin resistance constitutes the core pathophysiological foundation of metabolic syndrome, serving not only as the underlying basis for various chronic metabolic disorders—including type 2 diabetes mellitus, non-alcoholic fatty liver disease, hypertension, dyslipidemia, atherosclerotic cardiovascular disease, and polycystic ovary syndrome—but also as a pathological state necessitating timely intervention (1). From an epidemiological perspective, the World Health Organization categorized obesity as one of the five leading global mortality risk factors in 2009, with an estimated 70–80% of chronic diseases being associated with obesity. Research indicates that the standardized prevalence rate of insulin resistance among adults aged ≥25 years reaches as high as 29.22%, a proportion substantially exceeding that observed in healthy populations (2, 3). In recent years, the incidence of abdominal obesity with concomitant insulin resistance has continued to escalate in parallel with the rising obesity rate: the incidence of insulin resistance (IR) in the general population has reached 18%, with prevalence rates among obese individuals (29.19%) significantly higher than those observed in non-obese individuals (8.4%) (4). As the trajectory of obesity with IR accelerates, the burgeoning patient population imposes considerable strain on chronic disease prevention and treatment efforts. Addressing the prevention and treatment of abdominal obesity with IR is therefore imperative, driven by comprehensive considerations encompassing disease burden, economic implications, and public health significance (5). Notably, insulin resistance is reversible through active weight reduction and lifestyle modifications, thereby offering a critical window for the prevention of type 2 diabetes mellitus (6). Consequently, abdominal obesity with concomitant insulin resistance is regarded as a pivotal period for early preventive intervention against chronic metabolic diseases (7). This underscores the urgent necessity to identify effective and safe interventions capable of ameliorating abdominal obesity with insulin resistance, reducing obesity rates and the incidence of chronic metabolic disorders, and ultimately enhancing the quality of life of affected patients.

Currently, intervention strategies for abdominal obesity with concomitant insulin resistance primarily encompass lifestyle modifications, pharmacotherapy, and metabolic surgery (1). However, each of these approaches presents varying degrees of limitations: although lifestyle interventions serve as the foundational approach, poor patient adherence frequently results in weight regain; pharmacotherapy faces restricted clinical application due to its potential adverse effects (e.g., edema and increased fracture risk); and while metabolic surgery demonstrates considerable efficacy, its indications are largely confined to individuals with severe obesity, accompanied by risks of complications including hemorrhage and infection (8).

Currently, acupuncture-based external therapies, as complementary and alternative approaches, have garnered increasing attention. Commonly employed clinical interventions include manual acupuncture, electroacupuncture, acupoint catgut embedding, and acupoint application, with studies confirming their potential in improving anthropometric parameters and metabolic profiles (9). Among these, acupoint catgut embedding (ACE), as a distinctive acupuncture technique, involves the implantation of absorbable catgut sutures into specific acupoints, thereby achieving sustained stimulation for 2–4 weeks. Compared with conventional acupuncture, its prolonged stimulation mechanism significantly reduces treatment frequency, thereby enhancing patient adherence (10). Evidence suggests that ACE combined with lifestyle interventions is superior to lifestyle modifications alone or conventional pharmacotherapy in reducing body weight, body mass index, waist circumference, and improving the homeostasis model assessment of insulin resistance (HOMA-IR) index (11). A comprehensive review encompassing 18 clinical trials and 25 animal studies has proposed that acupuncture may serve as a first-line therapeutic strategy for insulin resistance (12). Acupoint catgut embedding has been shown to reduce serum leptin levels, increase leptin receptor expression in the hypothalamus, enhance the biological effects of leptin, alleviate leptin resistance in obese individuals, modify adipose tissue morphology, and regulate lipid profiles, thereby achieving weight reduction (13); these effects may be associated with modulation of the neuro-endocrine-immune network. Furthermore, acupoint catgut embedding ameliorates insulin resistance and exerts weight-reducing effects by downregulating the protein and mRNA expression of inducible nitric oxide synthase—a marker of M1-type macrophages—in epididymal adipose tissue, while upregulating arginase expression, a marker of M2-type macrophages, thereby regulating macrophage polarization and inhibiting the release of inflammatory cytokines from adipose tissue (14).

Although accumulating evidence supports the therapeutic potential of acupoint catgut embedding (ACE), its comparative efficacy remains unclear. A 2022 network meta-analysis on acupuncture for obesity with insulin resistance included only five trials, limiting its conclusions (11), and did not comprehensively compare ACE with lifestyle interventions, pharmacotherapy, or other acupuncture techniques. To date, no network meta-analysis has specifically focused on ACE for abdominal obesity with concomitant insulin resistance. Moreover, existing systematic reviews have often pooled heterogeneous protocols without adequately exploring key sources of clinical heterogeneity—such as embedding depth, suture materials, or baseline insulin resistance severity. This limitation is particularly relevant given the well-established ethnic differences in obesity phenotypes (with distinct waist circumference thresholds for Asian and Western populations) and the absence of universally accepted HOMA-IR cutoffs for defining insulin resistance severity. Consequently, the current evidence lacks the granularity to identify which patient subgroups—defined by ethnicity or metabolic severity—are most likely to benefit from ACE, or to determine its optimal technical parameters. These gaps underscore the need for a more nuanced investigation to inform personalized therapeutic strategies. In medical research, conventional meta-analysis is confined to direct comparisons between two interventions, thereby limiting its analytical scope. In contrast, network meta-analysis represents an innovative approach that integrates both direct and indirect evidence. This methodology enables researchers to synthesize data from multiple randomized controlled trials, estimate the probability of each intervention’s relative efficacy, and ultimately rank and compare different therapeutic strategies (15, 16). Leveraging this methodological advancement, we will conduct a systematic review and network meta-analysis to evaluate the efficacy and safety of acupoint catgut embedding for abdominal obesity with concomitant insulin resistance. By quantitatively assessing its relative advantages and potential benefits, this study aims to provide evidence-based clinical guidance for both clinicians and patients to inform treatment decision-making.

## 2 Methods

### 2.1 Objectives

1. Efficacy Evaluation: This systematic review and network meta-analysis will quantitatively assess the efficacy of acupoint catgut embedding in reducing HOMA-IR among patients with abdominal obesity and insulin resistance, to determine optimal strategies for improving insulin sensitivity and metabolic outcomes. Primary outcome is HOMA-IR; secondary outcomes include fasting glucose, fasting insulin, waist circumference, body mass index, lipid profile, and inflammatory markers. Network meta-analysis will establish comparative efficacy rankings, thereby informing clinical decision-making.
2. Safety Evaluation: This study will assess the incidence of adverse events (AEs) associated with different acupoint catgut embedding modalities in the treatment of abdominal obesity with insulin resistance, thereby establishing a comprehensive safety profile. AEs will be systematically documented and analyzed, encompassing local complications—including pain, bleeding, hematoma, infection, foreign body reaction, catgut allergy or rejection, and keloid formation—as well as systemic reactions such as fever, needle fainting, and vasovagal responses. In line with current clinical practice and published evidence, high-frequency ultrasound will be employed when clinically indicated to confirm the diagnosis of hematoma, deep infection, or subcutaneous induration, given its established utility in differentiating these conditions from nonspecific inflammatory changes. Subgroup analyses based on embedding depth (adipose, muscular, or alternating layers), catgut specifications, and treatment frequency will be performed to identify factors influencing safety, thereby informing the development of optimized protocols that balance efficacy with safety.

### 2.2 Research registration

This systematic review protocol was rigorously developed in full accordance with the Preferred Reporting Items for Systematic Reviews and Meta-Analyses Protocols (PRISMA-P) guidelines (17) and the PRISMA extension for network meta-analyses (18). To ensure methodological transparency and reproducibility, the protocol has been registered prospectively in the International Prospective Register of Systematic Reviews (PROSPERO; registration number: CRD420261331031). The completed PRISMA-P checklist is provided as Supplementary Appendix S1.

### 2.3 Search strategy

A comprehensive search strategy will be employed, combining Medical Subject Headings (MeSH) and free-text terms to capture studies related to acupoint catgut embedding, abdominal obesity, insulin resistance, and the homeostasis model assessment of insulin resistance (HOMA-IR). Boolean operators (OR, AND) will be used to refine the search. The search protocol is designed to include all available literature from the inception of each database until 1 March 2026. The following electronic databases will be systematically searched: PubMed, Embase, the Cochrane Library, Web of Science, Scopus, ClinicalTrials.gov, the WHO International Clinical Trials Registry Platform (WHO-ICTRP), OpenGrey, ProQuest, China National Knowledge Infrastructure (CNKI), VIP Database, WanFang Database, and the Chinese Biomedical Literature Database (CBM). Additionally, the reference lists of all included studies, along with relevant clinical trial reports and review articles, will be manually screened. A manual search will also be conducted through specialized journal collections, bibliographies, and conference proceedings focusing on acupoint catgut embedding therapy, obesity, and insulin resistance. The detailed search strategies for both Chinese and English databases are provided in Supplementary Appendix S2.

### 2.4 Study selection criteria

#### 2.4.1 Types of participants

The study population will consist of individuals diagnosed with abdominal obesity and insulin resistance according to established criteria. Abdominal obesity will be defined by standardized waist circumference thresholds (e.g., ≥90 cm for Asian men, ≥85 cm for Asian women, or ethnicity-specific cutoffs) or waist-to-hip ratio, following IDF, NCEP-ATP III, or other consensus guidelines. Insulin resistance will be defined as HOMA-IR exceeding a population-specific threshold (e.g., >2.68 for Chinese individuals), with or without impaired fasting glucose or glucose intolerance. Studies on metabolic syndrome that include these core components will also be eligible. No restrictions will be applied regarding sex, age, occupation, or education level. Individuals with secondary obesity or insulin resistance caused by endocrine disorders (e.g., Cushing’s syndrome, hypothyroidism) or drug-induced metabolic disturbances (e.g., long-term glucocorticoid use) will be excluded.

#### 2.4.2 Types of interventions

The intervention group must receive acupoint catgut embedding therapy. Eligible ACE interventions will be characterized by detailed extraction of suture material type and specifications (e.g., PGLA, catgut), embedding technique (e.g., incision method, syringe method, guide tube method), embedding depth (subcutaneous, adipose layer, muscular layer, or mixed layer), acupoint selection protocol (standardized or individualized), treatment regimen (frequency, duration, suture retention period), and auxiliary instruments (e.g., ultrasound guidance). To isolate the specific effect of acupoint catgut embedding, co-interventions are permitted only when administered equivalently across both groups. Additionally, studies permitting concomitant interventions will be eligible for inclusion only when such designs enable the isolation of the specific effect attributable to acupoint catgut embedding. No restrictions will be applied regarding treatment duration or follow-up period.

#### 2.4.3 Types of control groups

For the control group, a three-tiered hierarchical framework was established based on the network connectivity requirements of the network meta-analysis, aiming to minimize clinical heterogeneity and ensure analytical validity. We explicitly distinguish between ACE as an add-on therapy (ACE plus lifestyle intervention versus lifestyle intervention alone) and ACE as an independent intervention (ACE versus no treatment, sham/placebo, or other active interventions). The first tier permits inactive comparators, including sham embedding/placebo—defined as non-insertive procedures, superficial needling without suture implantation, or insertion of inert material mimicking catgut embedding without therapeutic effect—as well as blank control (no treatment or waitlist). The second tier allows active comparators comprising guideline-recommended lifestyle interventions (diet, exercise, or behavioral therapy) and pharmacotherapy (e.g., metformin, thiazolidinediones, GLP-1 receptor agonists, or other insulin sensitizers). The third tier includes other acupuncture modalities (e.g., manual acupuncture, electroacupuncture, auricular acupuncture, acupoint application, or acupressure) as active controls, provided they are included only when direct comparative evidence is available to maintain network connectivity. Studies evaluating combination therapy—comparing acupoint catgut embedding plus an intervention versus the same intervention alone—are eligible across all tiers. Trials with multiple control arms will be included, with all relevant pairwise comparisons considered within the network meta-analysis framework. No restrictions will be imposed regarding treatment duration or follow-up period.

#### 2.4.4 Types of outcomes

##### 2.4.4.1 Primary outcomes

The primary outcome is improvement in insulin sensitivity following acupoint catgut embedding for abdominal obesity with insulin resistance, measured by the homeostasis model assessment of insulin resistance (HOMA-IR) index. HOMA-IR is calculated as [fasting glucose (mmol/L) × fasting insulin (μU/mL)]/22.5. This index integrates fasting glucose and insulin levels to reflect hepatic and peripheral insulin sensitivity and is the most commonly employed surrogate marker for insulin resistance in clinical research (19). Higher HOMA-IR values indicate greater insulin resistance, with a threshold of ≥2.68 typically defining insulin resistance in Asian populations.

##### 2.4.4.2 Secondary outcomes

Secondary outcomes include metabolic, anthropometric, and safety parameters: fasting glucose and insulin, lipid profiles (triglycerides, total cholesterol, HDL, LDL), inflammatory markers (TNF-*α*, IL-6, CRP), waist circumference, waist-to-hip ratio, body mass index, visual analogue scale for appetite, and dietary diaries. Adverse events will be recorded and analyzed to assess modality-specific safety, including local complications (pain, bleeding, hematoma, infection, foreign body reaction, catgut allergy/rejection, keloid formation) and systemic reactions (fever, needle fainting, vasovagal responses). These measures provide comprehensive assessment of glycemic control, lipid metabolism, anthropometric changes, and treatment safety.

#### 2.4.5 Types of studies

This review will include only published, peer-reviewed randomized controlled trials (RCTs), with no restrictions imposed on language or regional origin. Studies employing non-randomized designs, case–control studies, cohort studies, case reports, review articles preprints, and study protocols will be excluded from the analysis. Additionally, duplicate publications will be excluded, retaining only the study with the largest sample size in cases of population or dataset overlap.

### 2.5 Study selection

**Figure 1:**
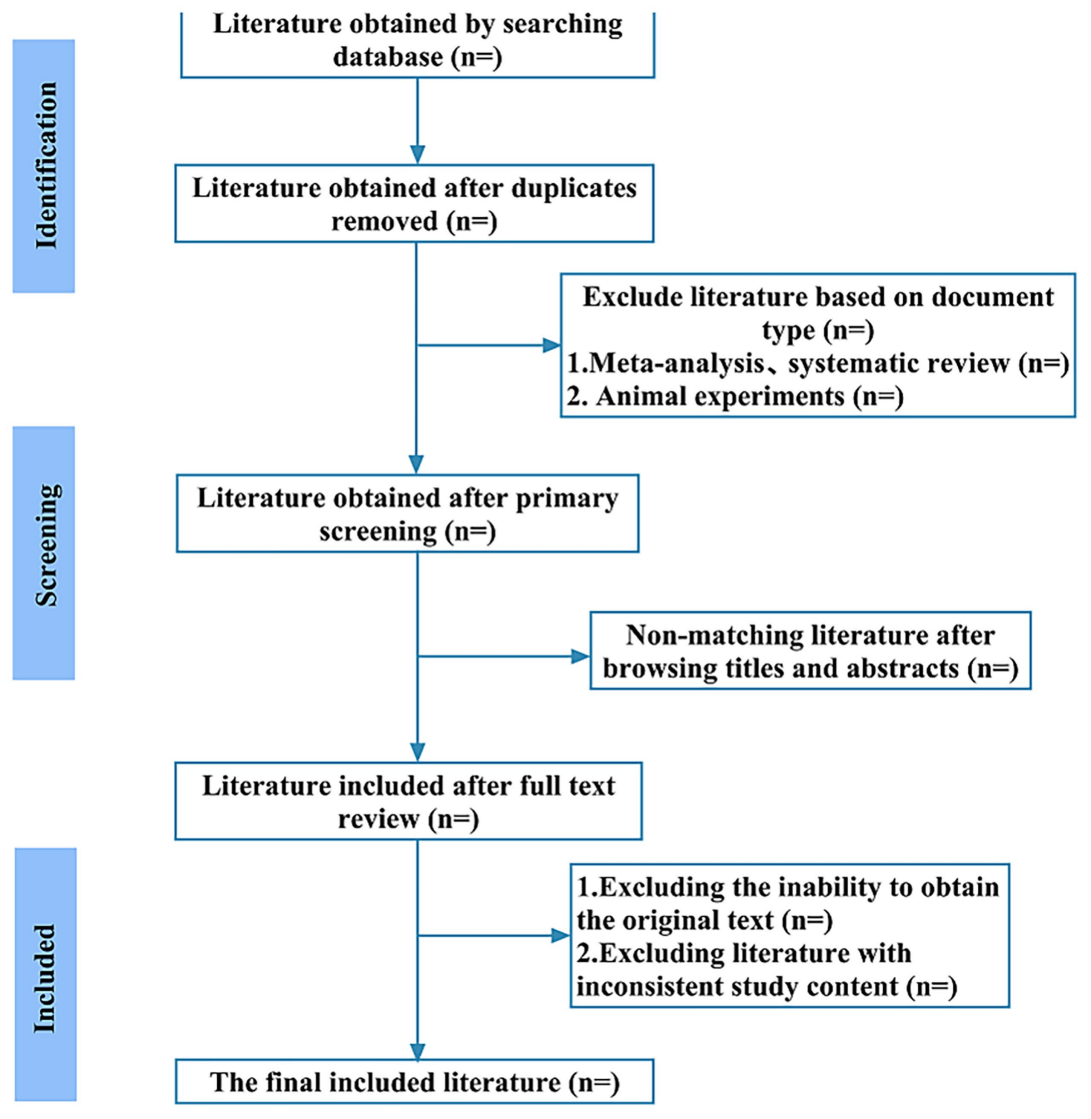
The details of the selection process.

In this study, two independent reviewers will independently perform literature searches and screening according to the search strategy. EndNote 20 will be used for reference management. After removing duplicates, the reviewers will sequentially screen titles, abstracts, and keywords, followed by a full-text assessment to identify literature meeting the inclusion criteria. Any disagreements will be resolved by a third reviewer. Reasons for exclusion will be systematically recorded, and the screening workflow is presented in the PRISMA flow diagram in Figure 1.

### 2.6 Data extraction

In this study, two independent data managers will extract relevant data from all finally included studies using standardized Excel forms. When insufficient or missing data are encountered during extraction, we will contact the corresponding authors via email to obtain complete information. All extracted data will undergo rigorous cross-checking prior to analysis. To ensure consistency and accuracy, the data extraction form will be pilot-tested on a sample of five included studies and refined accordingly by the review team. Any discrepancies will be discussed within the research team to ensure data accuracy. The extracted data will include the following:

*Study characteristics:* author, publication year, country, study design, study centers.

*Participant characteristics:* sample size, sex, age, diagnostic criteria, baseline characteristics (disease duration, obesity severity [BMI, waist circumference], baseline HOMA-IR); medication use; and treatment/follow-up duration.

*Intervention and control measures:* suture material type and specifications, embedding technique (e.g., incision method, syringe method, guide tube method), embedding location and depth, acupoint selection protocol, treatment duration, frequency, and course, technique method, and auxiliary instruments (e.g., ultrasound guidance).

*Outcome measures:* all reported outcomes from the included studies, quality of life assessments, records of adverse events, and time points of each measurement.

### 2.7 Risk of bias assessment

Two reviewers will assess the risk of bias for all included studies using the Cochrane Risk of Bias tool (RoB 2) (20). The RoB 2 tool evaluates five domains: bias arising from the randomization process, bias due to deviations from intended interventions, bias due to missing outcome data, bias in measurement of the outcome, and bias in selection of the reported result. Each domain is categorized as “low risk of bias,” “some concerns,” or “high risk of bias.” To minimize subjective discrepancies between reviewers, all reviewers received rigorous training based on detailed guidelines prior to the formal assessment. Any disagreements in the assessments were resolved through discussion with a third reviewer within the evaluation panel.

### 2.8 Data synthesis

#### 2.8.1 Pairwise meta-analysis

All statistical analyses will be performed using Stata software (Version 17.0; StataCorp LLC, College Station, TX, United States) and R software (Version 4.2.0; R Foundation for Statistical Computing, Vienna, Austria). The homeostasis model assessment of insulin resistance (HOMA-IR) value will be treated as a continuous variable, with the mean difference (MD) and its 95% confidence interval (CI) employed as the summary effect size. If different studies utilize disparate measurement units or scales, the standardized mean difference (SMD) with its 95% CI will be applied. Heterogeneity will be quantified using the I^2^ statistic in conjunction with the *p*-value, with statistical significance defined as *p* < 0.05. When *I*^2^ ≤ 50%, a fixed-effects model will be adopted to estimate the pooled effect size. Conversely, when *I*^2^ > 50%, indicating substantial heterogeneity, a random-effects model will be employed for pooling. In such instances, further exploration of potential sources of heterogeneity will be undertaken, encompassing patient characteristics (e.g., age, baseline HOMA-IR levels, obesity severity), intervention features (e.g., embedding depth, treatment frequency, treatment course), and study design characteristics. The potential impact of these factors on the study findings will be elucidated in the discussion section.

#### 2.8.2 Network meta-analysis

We will perform a network meta-analysis within a frequentist framework using a random-effects model to combine direct and indirect evidence from all eligible interventions. Prior to analysis, we will verify that the evidence forms a connected network—a prerequisite for conducting network meta-analysis. For multi-arm trials, correlations between effect sizes will be accounted for using a multivariate meta-analysis approach.

The validity of indirect comparisons depends on the transitivity assumption, which requires that studies comparing different interventions be sufficiently similar in key clinical and methodological characteristics. To evaluate this assumption and investigate potential sources of heterogeneity, we will perform pre-specified subgroup analyses and meta-regression based on effect modifiers. These patient-level characteristics include obesity diagnostic criteria (reflecting ethnic differences), insulin resistance severity (stratified by HOMA-IR thresholds), age, body mass index, and baseline HOMA-IR levels. These factors, identified based on clinical experience, may influence treatment response and directly delineate the target population to which the findings apply, thereby serving as a foundation for determining optimal indications. For continuous outcomes, we will calculate mean differences (MD) or standardized mean differences (SMD), each presented with their 95% confidence intervals (CIs). The primary outcome, HOMA-IR, will be analyzed as a continuous variable. A network diagram will be constructed to visually present the evidence structure. In this diagram, nodes represent distinct interventions—such as acupoint catgut embedding, lifestyle intervention, pharmacotherapy, manual acupuncture, or electroacupuncture—with node size reflecting the total sample size for each intervention and edge thickness corresponding to the number of studies informing each direct comparison. Global inconsistency will be assessed using the design-by-treatment interaction model. A non-significant result (*p* > 0.05) will support the adoption of a consistency model for evidence synthesis. If significant inconsistency is detected (*p* < 0.05), we will examine network characteristics and the distribution of effect modifiers across studies to identify potential explanations. For any closed loops within the network, node-splitting analysis will be performed to evaluate local inconsistency by comparing direct evidence with indirect evidence derived from the rest of the network. If local inconsistency persists after investigation, sensitivity analyses or network meta-regression will be considered as appropriate.

Treatment rankings will be estimated using the surface under the cumulative ranking curve (SUCRA). Higher SUCRA values indicate a greater probability of superior efficacy in reducing HOMA-IR, and ranking distributions will be visualized through cumulative probability plots. We acknowledge that SUCRA rankings should be interpreted cautiously, particularly when the evidence network includes studies with high risk of bias or substantial heterogeneity. Therefore, SUCRA results will be presented alongside the certainty of evidence assessed by GRADE and will be complemented by sensitivity analyses to assess the robustness of rankings.

#### 2.8.3 Subgroup analysis

To assess potential sources of heterogeneity and support the transitivity assumption in network meta-analysis, we will perform subgroup analyses based on patient- and intervention-level characteristics. Patient-level factors include study population ethnicity or geographic region (e.g., Chinese vs. non-Chinese populations) to reflect differences in established HOMA-IR thresholds across ethnic groups, age, sex, and baseline body mass index. Intervention-level factors include embedding depth (adipose vs. muscular vs. mixed), treatment frequency (weekly, biweekly, monthly), duration (≤8 vs. >8 weeks), suture material (PGLA vs. catgut), and operational technique (comparing the incision method, which involves a small skin incision and suture, with the syringe method using a trocar needle for minimally invasive placement). Where data permit, we will also examine the impact of concomitant medication. These analyses aim to identify patient subgroups most likely to benefit from specific embedding protocols and inform individualized treatment decisions.

### 2.9 Sensitivity analysis

Sensitivity analysis will be performed using the leave-one-out method, sequentially excluding each study and subsequently reconstructing the model to calculate changes in the surface under the cumulative ranking curve (SUCRA) values for each intervention. A change in SUCRA value of less than 15% will indicate robust results. Additionally, if substantial heterogeneity (*I*^2^ > 50%) is detected in the preliminary analysis, meta-regression will be conducted to explore the relationship between study-level covariates—such as acupoint catgut embedding frequency, embedding depth, and treatment course—and effect sizes, thereby addressing heterogeneity and inconsistency, identifying influential studies with potentially high risk of bias, and validating the robustness and reliability of the findings. Furthermore, to assess the impact of diagnostic criteria heterogeneity, we will conduct a sensitivity analysis excluding studies that apply diagnostic criteria inconsistent with established population-specific norms. This will help determine whether diagnostic heterogeneity influences the overall findings and whether results remain robust when restricting to studies employing widely accepted, population-appropriate thresholds. In addition, to assess the stability of the SUCRA rankings, leave-one-out sensitivity analyses will be conducted by sequentially omitting each study and recalculating the corresponding SUCRA values. Rankings will be considered robust if the resulting values exhibit a relative change of less than 15% from the original estimates.

### 2.10 Publication bias

For any comparison with at least 10 eligible studies, we will assess publication bias using funnel plots, Egger’s regression test, and Begg’s test. Funnel plots—scatterplots of treatment effects against their standard errors—provide a visual assessment of asymmetry that may indicate publication bias or small-study effects. To complement this graphical evaluation, we will apply Egger’s linear regression test, which quantifies asymmetry by testing whether the regression intercept differs significantly from zero, and Begg’s rank correlation test, which examines the association between effect estimates and their variances. Both Egger’s and Begg’s tests have limited statistical power when fewer than 10 studies are available, a limitation we acknowledge. If asymmetry is detected, we will perform trim-and-fill analysis to estimate and adjust for the potential impact of missing studies. Together, these complementary methods provide a thorough evaluation of publication bias in our meta-analysis (21, 22).

### 2.11 Grading the quality of evidence

We assessed the certainty of evidence for each outcome using the Grading of Recommendations, Assessment, Development, and Evaluations (GRADE) framework (23). Evidence ratings were downgraded based on five predefined domains: risk of bias, inconsistency, indirectness, imprecision, and publication bias. The strength of evidence was categorized into four levels: high, moderate, low, and very low. High-quality evidence indicates that the true effect is likely close to the estimated effect and can be strongly recommended in decision-making for clinical practice. Conversely, very low-quality evidence suggests that the true effect is likely substantially different from the estimated effect (24). Given the inherent variability in acupuncture protocols—including acupoint selection, catgut material, embedding depth, and treatment frequency—as potential sources of clinical heterogeneity, intervention reporting quality will be assessed using the revised STRICTA checklist (25). This will characterize the acupuncture regimens and enable exploration of intervention-related heterogeneity in subsequent subgroup analyses or meta-regression.

### 2.12 Ethics and dissemination

Our study exclusively utilizes data obtained from publicly accessible databases and does not directly involve human subjects or public participation in any phase of the research. As all data are derived from previously published literature, this study does not require formal ethics approval from an institutional review board. The final research findings will undergo rigorous peer review and be published in reputable scientific journals.

## 3 Discussion

Abdominal obesity with insulin resistance, as a core pathological state of metabolic syndrome, is widely recognized as a key intervenable window for type 2 diabetes and cardiovascular disease. Non-pharmacological interventions serve as a cornerstone in its clinical management. Among these, acupoint catgut embedding—a widely used acupuncture modality—has demonstrated emerging potential in improving insulin sensitivity and metabolic parameters. Previous meta-analysis shows that catgut embedding at acupoint has a good effect on reducing weight and improving HOMA-IR. This study will comprehensively integrate the existing evidence through systematic review and network meta-analysis to evaluate the relative efficacy and safety of acupoint catgut embedding and related interventions in the treatment of abdominal obesity with insulin resistance. By synthesizing direct and indirect evidence, this protocol aims to provide evidence-based support for the effectiveness of acupoint catgut embedding in improving insulin sensitivity in patients with abdominal obesity and insulin resistance, thus providing a reference for clinical decision-making and intervention strategies. Although this study will strictly follow the PRISMA-P guidelines and the Cochrane Handbook methodology requirements, it may still face the following challenges and limitations in the implementation process: (1) Regional distribution bias: Research on acupoint catgut embedding is predominantly conducted in China, which may lead to geographical limitations in the findings. Therefore, caution should be exercised when extrapolating the results to other populations. We will comprehensively discuss the influence of this bias on the generalizability of the conclusions and suggest conducting multi-center international collaborative studies for future verification. (2) Challenges in defining interventions: There is significant clinical heterogeneity in the operation of acupoint catgut embedding, particularly regarding the lack of unified standards for embedding depth and materials This study will combine the literature and expert consensus to make an operational definition, but the results can only explain the efficacy within the framework of this definition, and cannot cover all clinical variants. (3) Heterogeneity in diagnostic criteria: The diagnostic criteria for abdominal obesity (waist circumference cutoffs: Asian criteria ≥90 cm for men, ≥85 cm for women; Western criteria ≥102 cm for men, ≥88 cm for women) and insulin resistance (HOMA-IR thresholds ranging from 2.5 to 2.68) vary, which may constitute a source of heterogeneity. To address this, we will perform subgroup analyses stratified by study population ethnicity or geographic region (e.g., Chinese vs. non-Chinese populations) and conduct sensitivity analyses by excluding studies using non-standard diagnostic criteria to evaluate their impact on the pooled effect estimates. (4) Publication bias risk: Positive results are more likely to be published, and Chinese-language literature may be particularly susceptible to publication bias. Although we will conduct systematic searches of both Chinese and English databases as well as gray literature, the potential influence of publication bias cannot be completely ruled out. When interpreting the results, we will discuss the findings in conjunction with funnel plots and Egger’s test results. (5) Methodological quality of primary studies: Included studies may have limitations such as inadequate description of randomization, unclear allocation concealment, difficulties in implementing blinding, and incomplete reporting of acupuncture interventions. We will assess the quality of intervention reporting using the STRICTA checklist, and the GRADE rating may be downgraded accordingly as a result of these methodological concerns. (6) Clinical heterogeneity: Variability in treatment duration, follow-up periods, obesity severity, and comorbidities may introduce clinical heterogeneity. Subgroup analyses and network meta-regression will be employed to explore potential sources of heterogeneity; however, due to limitations in the completeness of reporting in primary studies, some of these analyses may not be feasible. (7) Underreporting of adverse events: While evaluating the safety of ACE is a secondary objective, primary trials often lack standardized reporting of adverse events. This may hinder a comprehensive safety profile comparison within the network meta-analysis. (8) Interpretation of SUCRA rankings: SUCRA values may artificially favor certain interventions, particularly when the network includes studies at high risk of bias or with substantial heterogeneity. Accordingly, we will interpret these rankings cautiously, integrating GRADE assessments and sensitivity analyses. (9) Clinical heterogeneity of ACE: Acupoint catgut embedding (ACE) procedures vary considerably across studies in embedding depth, suture material, treatment frequency, and operational technique. Although we will explore these factors through prespecified subgroup analyses, such variability may inherently limit the comparability of pooled estimates. (10) HOMA-IR interlaboratory variability: Beyond the absence of universally accepted cutoffs, HOMA-IR values are subject to interlaboratory variability due to differences in assay methods and standardization, which may introduce additional heterogeneity. Where feasible, we will assess the potential impact through sensitivity analyses. (11) Heterogeneity of comparators: The network includes a diverse range of comparators, such as placebo, pharmacotherapy, lifestyle interventions, and other acupuncture modalities. While this broad inclusion enables comprehensive comparisons, it also introduces substantial clinical heterogeneity. We will explicitly distinguish between add-on and standalone ACE interventions in the network structure and discuss the interpretability of cross-category comparisons.

## Data Availability

The original contributions presented in the study are included in the article/Supplementary material, further inquiries can be directed to the corresponding author.
