# Supplementary material for: Acupoint catgut embedding for improving HOMA-IR in patients with abdominal obesity and insulin resistance: a protocol for systematic review and network meta-analysis
Source: Front Nutr. 2026 May 8;13:1828235. doi: 10.3389/fnut.2026.1828235 (PMC13218076; doi:10.3389/fnut.2026.1828235)
Supplement: Supplementary file 2 [file Table_2.docx]

| Order | Strategy |
| --- | --- |
| #1 | Search: “Obesity, Abdominal”[Mesh] |
| #2 | Search: “Insulin Resistance”[Mesh] |
| #3 | Search: (abdominal obesity[Title/Abstract]) OR (central obesity[Title/Abstract]) OR (visceral obesity[Title/Abstract]) OR (android obesity[Title/Abstract]) OR (waist circumference[Title/Abstract]) |
| #4 | Search: (insulin resistance[Title/Abstract]) OR (IR[Title/Abstract]) OR (HOMA-IR[Title/Abstract]) OR (HOMA IR[Title/Abstract]) OR (homeostasis model assessment[Title/Abstract]) OR (insulin sensitivity[Title/Abstract]) |
| #5 | #1 OR #3 |
| #6 | #2 OR #4 |
| #7 | #5 AND #6 |
| #8 | Search: "Acupuncture Therapy"[Mesh] |
| #9 | Search: (acupoint catgut embedding[Title/Abstract]) OR (catgut embedding therapy[Title/Abstract]) OR (acupoint embedding[Title/Abstract]) OR (catgut implantation[Title/Abstract]) OR (catgut embedding at acupoint[Title/Abstract]) OR (acupoint catgut implantation[Title/Abstract]) OR (catgut embedding[Title/Abstract]) |
| #10 | #8 OR #9 |
| #11 | Search: (randomized controlled trial[Publication Type]) OR (randomized[Title/Abstract]) OR (randomly[Title/Abstract]) OR (trial[Title/Abstract]) OR (RCT[Title/Abstract]) OR (placebo[Title/Abstract]) |
| #12 | #7AND #10 AND #11 |

Supplementary Material

# S2 The search strategy for PubMed

**The search strategy for China National Knowledge Infrastructure (CNKI)**

| Strategy |
| --- |
| SU%='腹型肥胖'+'中心性肥胖'+'内脏肥胖'+'腹部肥胖'+'向心性肥胖'+'腰围' AND SU%='胰岛素抵抗'+'IR'+'HOMA-IR'+'胰岛素敏感性'+'胰岛素抵抗指数' AND SU%='穴位埋线'+'埋线疗法'+'羊肠线埋植'+'埋线'+'穴位植入' AND TKA%='随机对照试验'+'随机对照'+'随机分组'+'随机'+'RCT'+'临床试验' |
